# Editorial: Artificial intelligence and big data for value-based care

**DOI:** 10.3389/fmed.2023.1134021

**Published:** 2023-01-23

**Authors:** Cheng Ta Han, Ming-Chin Lin, Abeer Alsadoon, Md. Mohaimenul Islam

**Affiliations:** ^1^Department of Neurosurgery, Shuang Ho Hospital, Taipei Medical University, New Taipei City, Taiwan; ^2^Taipei Neuroscience Institute, Taipei Medical University, Taipei, Taiwan; ^3^Graduate Institute of Biomedical Informatics, College of Medical Science and Technology, Taipei Medical University, Taipei, Taiwan; ^4^School of Computing and Mathematics, Charles Sturt University (CSU), Wagga Wagga, NSW, Australia; ^5^International Center for Health Information Technology, College of Medical Science and Technology, Taipei Medical University, Taipei, Taiwan

**Keywords:** artificial intelligence, healthcare, healthcare system, value-based care, healthcare cost

Quality care is a key component of the health care system. The gap between actual care received and ideal care quality in the existing healthcare system is enormous. Although healthcare spending is remarkably rising than any other global economy, the healthcare system is still facing immense challenges in inaccurate diagnoses, medication errors, inappropriate or unnecessary treatments, and insufficient clinical practices. The World Health Organization (WHO) report 2020 shows that global spending on healthcare has reached US$ 8.3 trillion, ~10% of the global GDP (1). A decisive effort is necessary to move value-based care from fee-for-service to improve financial and clinical performance. Value-based care has the potential to promote better clinical outcomes without increasing costs.

A shift to value-based care from fee-for-service is not a dream because of the availability of patient data in the electronic health record (EHR) systems, standardization framework, and advanced algorithms. Clinicians can collect overwhelming amounts of patient data and utilize advanced analytical tools to make accurate predictions and actionable insights to improve overall provider performance, decrease medical errors, and reduce healthcare waste (2). Chen et al. developed an artificial intelligence (AI) system to correctly classify long-term cardiovascular outcomes in patients with normal ejection fraction. Echocardiographic data from 61,525 patients were collected to develop an AI model, which was later internally and externally validated using data from 3,810 and 5,760 patients. This AI-based system was able to stratify patients with a left ventricular end-diastolic diameter (LV-D) and predicts ECG-EF accurately with high AUCs. Nowadays, stereotactic body radiotherapy (SBRT) is considered one of the key treatment options for patients with early-stage lung cancer. It has shown a beneficial effect in improving tumor control and overall survival rate. A recent study tested the performance of the Mask R-CNN-based algorithm for evaluating the dose accuracy of a lung SBRT treatment plan with the target of a newly predicted internal target volume (ITV_predict_) and the feasibility of its clinical application (Zhang et al.). The cone-beam CT (CBCT) images were collected from early-stage 45 lung cancer patients who underwent SBRT at Huadong Hospital. This AI-enable tool was able to predict the ITV volume of large tumors more accurately, which ensures the feasibility of this automated model in making an appropriate treatment plan.

Alzheimer's disease (AD) is a critical global health problem contributing to a substantial financial burden. A previous study reported that ~6.5 million aged 65 or older are living with AD in the USA. Early identification of AD patients significantly reduces healthcare costs and improves patients' quality of life. Since AI techniques based on MRI are being used in the early diagnosis of AD, a novel deep learning radiomics (DLR) model was developed to classify cognitively normal adults at risk of AD from normal control using T1-weighted structural MRI images (Jiang et al.). A total of 417 patients were included in the study, and MRI data of those patients were used to divide patients into pre-AD (181 individuals) and control groups (236 individuals) based on a standard uptake ratio >1.18. AI model achieved state-of-the-art performance in classifying pre-AD and normal control with an accuracy of 89.85% ± 1.12%. It is now established that advanced AI algorithms have surpassed traditional statistical methods in image recognition and being extensively used in medical image analysis. In the last decade, AI-based radiomic models have made meaningful contributions to detecting chronic diseases, including lung cancer. A systematic review and meta-analysis included a total of 19 published studies to evaluate the diagnostic accuracy of AI models for lung cancer staging (Zheng et al.). The findings of AI models have the potential to improve diagnostic accuracy for lung cancer staging in terms of sensitivity, specificity, and the area under the receiver operating curve (AUROC).

AI models have tremendous potential to reduce medical errors, effectively utilize limited resources, and ultimately improve value by making accurate and effective clinical decisions. Recently, several studies internationally validated AI tools and achieved classification accuracy performance that outperformed human performance (3, 4). Transforming to value-based care from a fee-for-service will face significant challenges in achieving quality for all and will take time. But widespread adoption of value-based care would help lower healthcare costs while simultaneously improving the quality of care.

## Author contributions

All authors listed have made a substantial, direct, and intellectual contribution to the work and approved it for publication.
